# Time course of liver mitochondrial function and intrinsic changes in oxidative phosphorylation in a rat model of sepsis

**DOI:** 10.1186/s40635-018-0197-y

**Published:** 2018-09-05

**Authors:** Pierre Eyenga, Damien Roussel, Jerome Morel, Benjamin Rey, Caroline Romestaing, Virginie Gueguen-Chaignon, Shey-Shing Sheu, Jean Paul Viale

**Affiliations:** 1 Service de réanimation, centre hospitalier de Sens, 1 avenue pierre de Coubertin, 89100 Sens, France; 2 CNRS, UMR 5023, Laboratoire d’Ecologie des Hydrosystèmes Naturels et Anthropisés, Université Claude Bernard Lyon 1, 69622 Villeurbanne, France; 3 Protein Science Facility, Institut de Biologie et Chimie des Protéines, CNRS Université Claude Bernard Lyon 1, 69007 Lyon, France; 4 Center for Translational Medecine, Thomas Jefferson University, Philadelphia, USA; 5 CNRS, UMR 5558, Laboratoire de biométrie et de biologie évolutive, Université Claude Bernard Lyon 1, 69622 Villeurbanne, France; 6 Service de réanimation chirurgicale, CHU de Saint Etienne, 42000 Saint Etienne, France; 7 Université Claude Bernard Lyon, 69008 Lyon, France

**Keywords:** Severe sepsis, Proinflammatory cytokines, Mitochondria, Oxidative phosphorylation, Oxidative stress, Cytochrome c oxidase, ATP synthase, Uncoupling protein 2, Biogenesis factors

## Abstract

**Background:**

Tissue ATP depletion and oxidative stress have been associated with the severe outcomes of septic shock. One of the compensatory mechanisms to alleviate the sepsis-induced mitochondrial dysfunction could be the increase in oxidative phosphorylation efficiency (ATP/O). We propose to study liver mitochondrial function and oxidative stress and the regulatory mechanism of mitochondrial oxidative phosphorylation efficiency in an animal model of sepsis.

**Methods:**

We induced sepsis in rats by cecal ligation and perforation (CLP). Six, 24, or 36 h following CLP, we measured liver mitochondrial respiration, cytochrome c oxidase activity, and membrane permeability. We determine oxidative phosphorylation efficiency, by measuring ATP synthesis related to oxygen consumption at various exogenous ADP concentrations. Finally, we measured radical oxygen species (ROS) generation by liver mitochondria and mRNA concentrations of UCP2, biogenesis factors, and cytokines at the same end points.

**Results:**

CLP rats presented hypotension, lactic acidosis, liver cytolysis, and upregulation of proinflammatory cytokines mRNA as compared to controls. Liver mitochondria showed a decrease in ATP synthesis and oxygen consumption at 24 h following CLP. A marked uncoupling of oxidative phosphorylation appeared 36 h following CLP and was associated with a decrease in cytochrome c oxidase activity and content and ATP synthase subunit β content (slip mechanism) and an increase in mitochondrial oligomycin-insensitive respiration, but no change in mitochondrial inner membrane permeability (no leak). Upregulation of UCP2 mRNA resulted in a decrease in mitochondrial ROS generation 24 h after the onset of CLP, whereas ROS over-generation associated with slip at cytochrome c oxidase observed at 36 h was concomitant with a decrease in UCP2 mRNA expression.

**Conclusions:**

Despite a compensatory increase in mitochondrial biogenesis factors, liver mitochondrial functions remain altered after CLP. This suggests that the functional compensatory mechanisms reported in the present study (slip at cytochrome c oxidase and biogenesis factors) were not strong enough to increase oxidative phosphorylation efficiency and failed to limit liver mitochondrial ROS over-generation. These data suggest that treatments based on cytochrome c infusion could have a role in mitochondrial dysfunction and/or ROS generation associated with sepsis.

**Electronic supplementary material:**

The online version of this article (10.1186/s40635-018-0197-y) contains supplementary material, which is available to authorized users.

## Background

Sepsis and septic shock remain a main cause of death in intensive care units despite numerous therapeutic trials [1]. If they survive the early shock phase [2], most patients die from sepsis-induced multiple organ failure [3]. Although liver failure occurs after pulmonary and renal insufficiencies [4, 5], it had been shown that hepatocellular dysfunction occurs early in the course of sepsis and persists during the late hypodynamic stage of sepsis [6–8]. The early depression of hepatocellular function has been ascribed to a liver microcirculatory alteration or to a release of proinflammatory cytokines [8]. Whereas the link between the initial inflammatory activation and the hepatocellular dysfunction leading to a liver failure remains elusive, mitochondrial dysfunctions are thought to play a major role. Mitochondrial dysfunctions are associated with severe abnormalities in tissue oxygen extraction and utilization [9, 10] which may ultimately lead to a critical depletion of cellular ATP and thus sepsis-mediated injury [11]. Due to an early downregulation of glycolysis pathways [12–14], cellular energetic adaptation to septic shock requires an increase in mitochondrial oxidative phosphorylation activity and/or efficiency (i.e., the amount of ATP produced per atom of oxygen consumed) to maintain mitochondrial ATP generation [15] and prevent bioenergetic failure and death. To adjust mitochondrial activity to ATP demand in stress conditions such as septic shock, the activity and/or efficiency of oxidative phosphorylation have to be fine-tuned [16]. This fine-tuning involves the control of (i) the inner mitochondrial membrane permeability (which regulates proton leakage), (ii) the activity and/or content of the mitochondrial respiratory chain proteins and ATP synthase, as well as (iii) the mechanisms preventing over-generation of mitochondrial reactive oxygen species (ROS). Among these steps, changes in the cytochrome c oxidase stoichiometry and/or activity [17, 18], the proton leak activity or the transcription of the UCP2 gene, and an increase in mitochondrial non-coupled respiration with phosphorylation are thought to play special roles [19, 20].

Potential mechanisms of mitochondrial dysfunction consecutive to sepsis have been proposed [9, 21, 22]. However, the intrinsic changes occurring in mitochondrial oxidative phosphorylation during sepsis that would maintain or improve mitochondrial ATP production while decreasing ROS generation have not been described. We present evidence of liver mitochondrial dysfunction and some data regarding the efficiency of oxidative phosphorylation. As the increase in mitochondrial respiration non-coupled with phosphorylation and expression of UCP2 may play a role in controlling ROS generation and in preserving mitochondrial functions, we have measured their activation as well.

## Methods

The animal bioethics board of the Université Claude Bernard de Lyon approved this experimental study with license number BH 2009-01. The “Principles of laboratory animal care” (NIH publication No. 86-23, revised 1985) were followed.

### Surgical procedure

#### Animal

Fifty-four male Wistar rats were obtained from IFFA-CREDO (Arbresle, France) and maintained at 23 ± 1 °C under an artificial 12-h day-night cycle (12 h light-dark cycle). Rats were fed a standard diet and received water ad libitum and were allowed to adapt to laboratory conditions for at least 1 week. At the time of experiments, rats weighted 280–320 g.

#### Cecal ligation and puncture

Anesthesia and analgesia were induced using a combination of sodium pentobarbital (40 mg kg^−1^) and fentanyl (20 μg kg^−1^) administered intraperitoneally according to published protocols [23, 24]. Rats anesthetized with this anesthetic regimen exhibited surgical analgesia (no purposeful response to supramaximal noxious stimuli) with no evidence of cardio-respiratory dysfunctions. Rats were shaved in the abdomen and back, and then an abdominal mid-line incision (3 cm) was made under aseptic conditions in 30 animals to expose the cecum at the adjoining intestine. Half (50%) of the cecum was ligated tightly below the ileo-cecal junction without obstruction of the bowel. Three punctures were performed on the anti-mesenteric border using a 19-gauge needle, and gentle pressure was applied to the cecum to extrude a small amount of feces from the perforation sites. The whole bowel was returned into the abdominal cavity, and the abdominal incision was closed with sutures. Twelve of 30 cecal ligation and perforation (CLP) rats died between the 24th and 36th hours following CLP (40% mortality), so only 18 CLP rats entered the study successfully. Matching animals were randomly assigned to undergo a sham operation as the control group (*n* = 18) or serve as the untreated naïve group (0-h control, *n* = 6). Sham operation (control) involved laparotomy and cecal deliverance without ligation or puncture. All animals were resuscitated with subcutaneous bolus infusion of normal saline solution (10 mL kg^−1^) immediately after surgery and every 8 h. To alleviate discomfort, transdermic fentanyl (25 μg kg^−1^ h^−1^) was applied on the animal’s back throughout the postoperative period. Surviving CLP animals and matching sham-operated animals were compared with naïve untreated controls at 6, 24, and 36 h after surgery. At the end of each experimental period, the carotid artery was cannulated, and blood pressure was monitored for 1 h after 30 min of stabilization. Then, blood samples were collected by cardiac puncture for plasma analysis of lactic acid and alanine amino transferase (ALT). The liver was quickly dissected and stored in ice-cold isolation buffer for mitochondrial isolation and functional analysis (see below). A small piece of liver was immediately frozen in liquid nitrogen and stored at − 80 °C for molecular analysis.

### Lactic acid and alanine aminotransferase determination

Plasma ALT was measured by enzymatic reaction using commercial kits (Modular P Roche Diagnostics, Meylan, France), as were lactic acid (Hitachi 712-Roche Diagnostics Meylan France).

### Measurement of mRNAs in liver

Total RNA was extracted from liver using the TRIzol Reagent. Total RNA was treated with DNase I (Invitrogen, Carlsbad, CA, USA) according to the manufacturer’s recommendations. The integrity of the purified RNA was verified by agarose gel electrophoresis followed by ethidium bromide staining. Reverse transcription was performed using random hexamers as primers, reverse transcriptase (Superscript II), and 2 μg of total RNA in a total volume of 50 μL. The stability of expression levels under experimental and control conditions was investigated with the 18S ribosomal RNA (18S rRNA) gene as a housekeeping gene.

#### Real-time RT-PCR

The sequences of the PCR primers for IL-1β, TNF-α, UCP2, mTFA, NRF1, and 18S rRNA used in this study are listed in Additional file 1. PCR was performed using standard protocols with SyBR Green PCR Supermix as a fluorescent detection dye in an iCycler real-time PCR machine (Bio-Rad, Hercules, CA, USA). We used a two-step PCR amplification protocol with an annealing temperature of 60 °C for up to 40 cycles. Copy number was calculated with a standard curve obtained with known amounts of target DNA. 18S rRNA was used as an internal control for each sample. All PCRs for a given sample were performed in duplicate.

### Liver mitochondrial isolation

Liver mitochondria were prepared using a differential centrifugation protocol [25]. Briefly, liver was immediately dissected and cut up finely with sharp scissors and diluted 1:10 (*w*/*v*) in ice-cold isolation medium consisting of 250 mM sucrose, 20 mM Tris/HCl, and 1 mM EGTA, pH 7.3. The minced tissues were homogenized with a potter-Elvehjem homogenizer (three passages). The liver homogenate was centrifuged at 800 g for 10 min. The resulting supernatant was centrifuged at 1000×g for 10 min, filtered through cheesecloth, and recentrifuged at 8700×g for 10 min to pellet mitochondria. Finally, the pellet containing liver mitochondria was washed twice by suspension in the isolation buffer and centrifuged at 8700×g for 10 min. The protein concentration of mitochondrial suspensions was determined using the Biuret method with bovine serum albumin as a standard.

### Liver mitochondrial oxidative capacity and oxidative phosphorylation efficiency

#### Mitochondrial respiratory parameters

Maximal oxygen consumption was measured in a glass cell of 1.5 mL volume fitted with a Clark oxygen electrode (Rank Brothers Ltd., France) and thermostated at 37 °C. Mitochondria (1 mg of mitochondrial protein mL^−1^) were incubated in respiratory medium containing 120 mM KCl, 5 mM KH_2_PO_4_, 1 mM EGTA, 2 mM MgCl_2_, 0.3% of bovine serum albumin, and 3 mM HEPES, pH 7.4. Substrate concentrations were 5 mM succinate in the presence of 5 μM rotenone or pyruvate (5 mM) plus malate (2.5 mM). The active state of respiration (state 3) was initiated by the addition of 500 μmol ADP. To measure mitochondrial non-coupled respiration to phosphorylation, oligomycin (3 μg mL^−1^) was added. The fully active state of respiration was initiated by the addition of 2 μM carbonyl cyanide trifluoromethoxyphenylhydrazone (FCCP) in the presence of 3 μg mL^−1^ oligomycin. Thereafter, myxothiazol (3 μM) was added to fully inhibit succinate-supported respiration. Then, 2 mM ascorbate and 500 μM N,N,N’N′-tetra methyl-p-phenylenediamine (TMPD) were added, and the maximal respiration rate associated with isolated cytochrome c oxidase activity was recorded. The respiratory control ratio (RCR) refers to the ratio of oxygen consumed after adding ADP to that consumed in the presence of oligomycin.

#### Mitochondrial ATP synthesis and oxidative phosphorylation efficiency

To measure oxidative phosphorylation efficiency, oxygen consumption and ATP synthesis were performed at 37 °C in respiratory medium supplemented with glucose (20 mM), hexokinase (3 U mL^−1^) as previously described [25, 26]. Respiratory substrates were succinate (5 mM) in the presence of rotenone (5 μM) or pyruvate (5 mM) plus malate (2.5 mM). The mitochondrial ATP synthesis was initiated by the addition of four different amounts of ADP (100 μM, 20 μM, 5 μM, 1 μM). After recording the phosphorylating respiration rate (state 3) for 2 min, four 300 μL samples of mitochondrial suspension were withdrawn from the suspension every 30 s and were quenched in a perchloric acid solution consisting of 10% HClO_4_ and 25 mM EDTA. After centrifugation of the denatured protein (15,000×g for 6 min) and neutralization of the resulting supernatant, the ATP production was determined from the glucose-6-phosphate content of samples, which was assayed spectrophotometrically by monitoring the production of NADH in the presence of glucose-6-phosphate dehydrogenase at 340 nm [27]. Briefly, the supernatant was incubated in 1 mL of a reaction buffer consisting of NAD (0.5 mM), triethanolamine-HCl (50 mM), MgCl_2_ (7.5 mM), EDTA (3.75 mM), pH 7.4. The concentration of glucose-6-phosphate was calculated from the difference between the level of NADH measured before and 1 h after the addition of glucose-6-phosphate dehydrogenase (0.5 U). The rate of mitochondrial ATP production was calculated from the slope of the linear accumulation of glucose-6-phosphate over the sampling time interval (1.5 min). The linearity of glucose-6-phosphate accumulation allowed us to check that the system was in a steady state. It is important to note that we made sure that the ATP production rates were specific of the mitochondrial ATP synthase activity, by determining oxygen consumption and ATP synthesis rates in the presence of oligomycin (2 mg mL^−1^). Over the range of ADP concentrations used, a nonmitochondrial ATP synthesis activity was measurable at 100 mM.

### Liver mitochondrial membrane potential (ΔΨm) and oxygen consumption rate

The mitochondrial membrane potential was measured in a closed, stirred, Perspex chamber thermostated 37 °C and fitted with a Clark oxygen electrode (Rank Brothers Ltd). A combined triphenylmethylphosphonium (TPMP^+^) and reference electrodes were fitted through the lid. Liver mitochondria (1 mg of mitochondrial protein mL^−1^) were incubated in respiratory medium supplemented with 5 μM rotenone, 3 μg mL^−1^ oligomycin and 80 ng mL^−1^ nigericin. The TPMP^+^ electrode was calibrated by sequential 0.5 μM additions up to 2 μM TPMP^+^. The reaction was started by the addition of 5 mM succinate, and respiration was then gradually inhibited through successive steady states by addition of malonate up to 3 mM [28].

### Liver mitochondrial oxygen radical production

The rate of mitochondrial ROS production (H_2_O_2_) was measured at 37 °C in the respiratory medium following the rate of appearance of resorufin from Amplex Red with a Kontron SFM25 fluorescence spectrophotometer (excitation at 563 nm, emission at 587 nm) as described previously [25, 29]. Reaction conditions were 0.1 mg mL^−1^ of mitochondrial protein, 1 U mL^−1^ of horseradish peroxidase, and 10 μM Amplex Red. The reaction was initiated by the addition of succinate (5 mM) in the absence of rotenone. Calibration of H_2_O_2_ production was obtained by the addition of a known amount of H_2_O_2_.

### Western blot analysis

Cytochrome c oxidase subunit 1 (CcO1), and ATP synthase subunit β (ATP synthase β) were detected in liver homogenates by Western blot using standard techniques as described previously [25, 30]. The primary antibodies employed were a mouse anti-mouse polyclonal anti-CcO 1 (1:1000 dilution; Invitrogen, Cergy Pontoise, France), a mouse anti-mouse polyclonal anti-ATP synthase β (1:1000 dilution; Life Technologies, Courtaboeuf, France), and a rabbit anti-rabbit monoclonal anti-β-actin antibody (1:1000 dilution, Sigma Aldrich, Saint-Quentin, Fallavier, France). Horseradish peroxidase-linked goat anti-mouse or anti-rabbit secondary antibody (1:10,000 dilution; containing goat anti-mouse or anti-rabbit secondary antibody, Sigma Aldrich, Saint-Quentin, Fallavier France) was used for CcO1, ATP synthase β, and β-actin, as appropriate. The protein bands corresponding to CcO1, ATP synthase β, and β-actin (by molecular weight identification and specific positive immunoprevalence) were analyzed by means of density profile plots created using NIH ImageJ 1.63 software. Protein band density profiles for CcO1 and ATP synthase β were normalized for protein load by dividing by the matching density profiles for β-actin.

### Statistical analysis

Measured variables (e.g., ALT, lactic acid, H_2_O_2_ production, and mitochondrial function) were compared using two-way ANOVA (groups versus time). Differences between means were subsequently tested by Scheffe’s test. The level of mRNA expression and protein contents were compared using the non-parametric Kruskal-Wallis test (groups versus time). Differences between group means at specific time points were subsequently tested by the Mann-Whitney test. Values are presented as the mean ± SEM. Slopes between regressions were compared by a two-tailed *F* test. Statistical significance was recognized at *p* < 0.05.

## Results

### Biological parameters and inflammatory cytokine mRNA levels

**Fig. 1:**
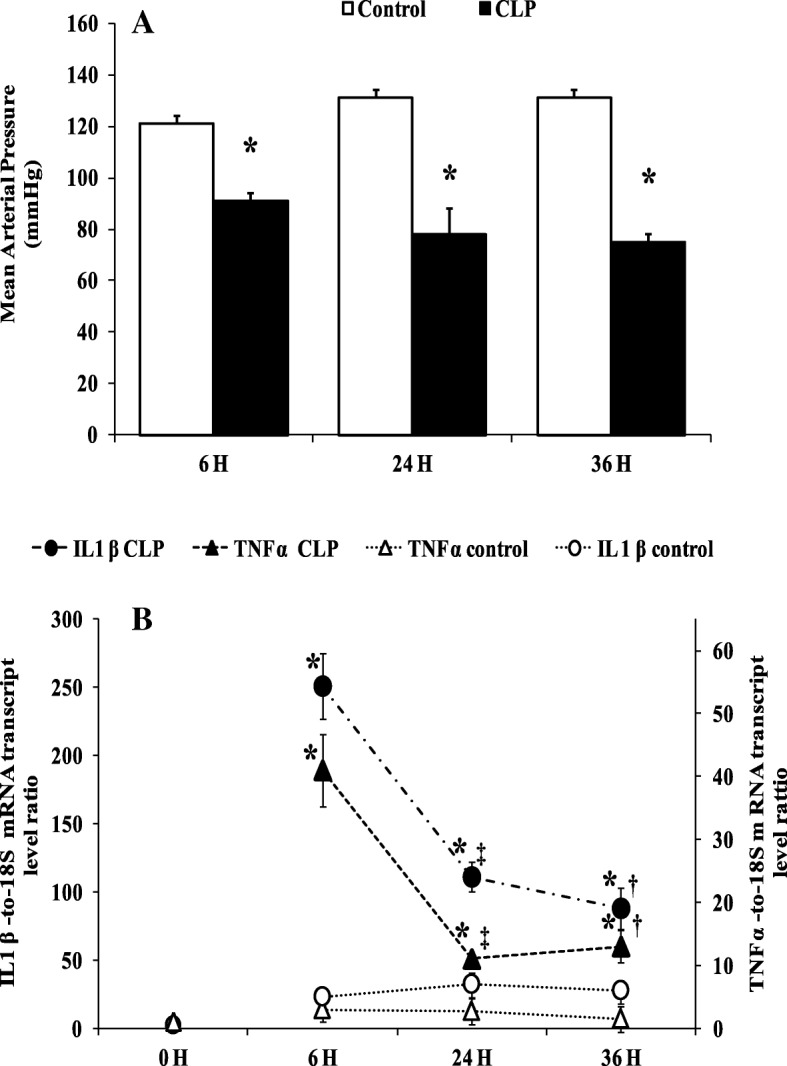
Mean arterial pressure and cytokines mRNA levels. **a** Mean arterial blood pressure (MAP mmHg) at the end time point in matching sham-operated control groups (white bars) and CLP groups (black bars). Data are means ± SEM. *p < 0.05 versus matching sham-operated control from six animals per group determined by surgical procedure and following period (0, 6, 24, 36 h). **b** TNF α and IL 1 β mRNA level were determined by real-time quantitative PCR in the liver of control groups (naïve and sham operated) (white symbols) and CLP groups (black symbols). Data are means ± SEM. *p < 0.05 versus control (matching sham operated and naïve); ‡*p* < 0.05 compared 6 h CLP and 24 h CLP; †*p* < 0.05 compared 6 h CLP and 36 h CLP, from five animals per group determined by surgical procedure and following period (0, 6, 24, 36 h)

Compared to the sham-operated control group, rats from the CLP groups showed hypotension (Fig. 1a), a higher plasma lactate, and hepatic dysfunction as evidenced by an increase in ALT at the time of sacrifice (Table 1). In these groups, the cytokine mRNA levels were higher than those of sham-operated control and naïve rats (control 0 h) (Fig. 1b). TNF-α mRNA was overexpressed 6 h after the onset of CLP (40.42 ± 5.71, *p* = 0.036, *n* = 5) compared to sham-operated control rats (2.58 ± 0.36, *n* = 5). At 24 h (8.28 ± 0.86, *n* = 5) and 36 h (11.30 ± 2.50, *n* = 5), the expression of TNF-α was reduced compared to that measured at 6 h (*p* = 0.044, *n* = 5) but remained higher than that of matching sham-operated controls at 24 h (2.71 ± 0.31) and 36 h (1.48 ± 0.23). A similar pattern was observed for IL-1β gene expression, with a higher expression observed at 6 h (257.80 ± 34.59, *p* = 0.0025, *n* = 5) compared to that at 24 h (111.23 ± 11.75, *p* = 0.0104, *n* = 5) and 36 h (88.10 ± 15.03, *p* = 0.0104, *n* = 5) in CLP rats (Fig. 1b).

**Table 1:** Plasma alanine amino transferase (ALT), and lactic acid in control and CLP groups over time. Results are means ± SEM

	6 h		24 h		36 h	
	CLP	Sham	CLP	Sham	CLP	Sham
ALT U/L	88.20 ± 9.88*	42.75 ± 5.94	172.60 ± 21.70*‡	43.00 ± 1.58	129.75 ± 15.93*	39.20 ± 2.59
Lactic acid mmol/L	4.25 ± 0.60	2.21 ± 0.53	6.29 ± 1.09*	2.71 ± 0.95	4.43 ± 0.73*	1.83 ± 0.34

*p < 0.05 compared with matching sham-operated control group; &*p* < 0.05 compared 24 and 36H CLP; ‡*p* < 0.05 compared 24 and 6H CLP, from 6 animals per group determined by surgical procedure and following period (6, 24, 36 h)

### Effect of sepsis on mitochondrial oxidative phosphorylation

**Fig. 2:**
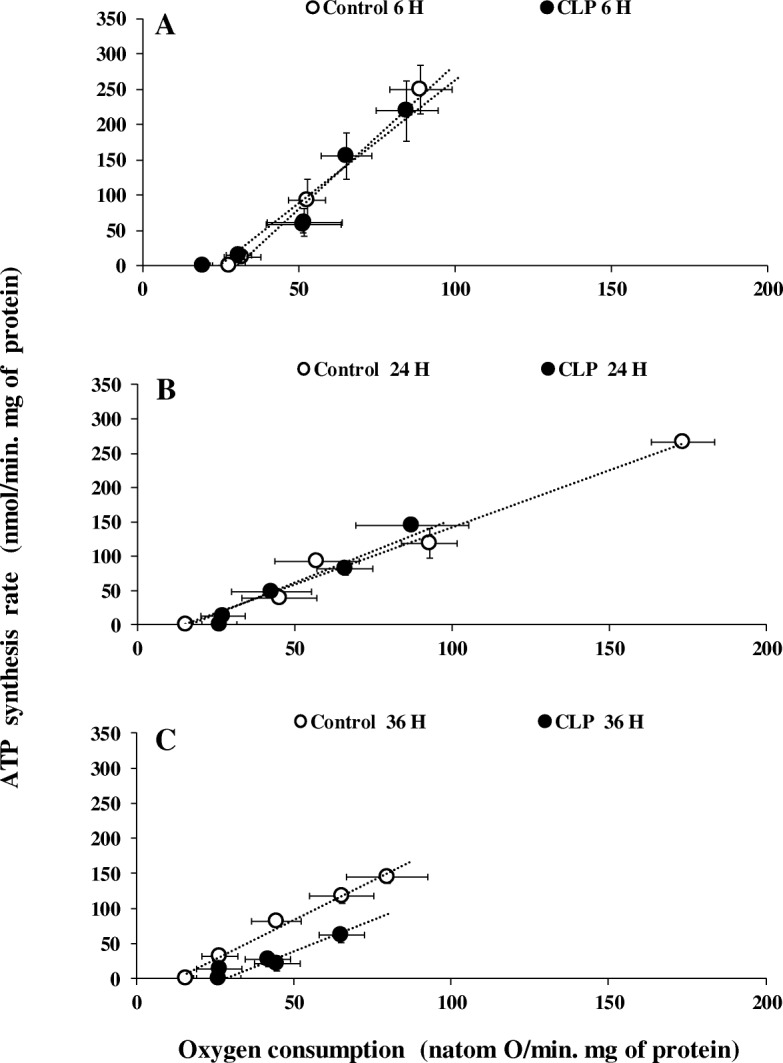
Mitochondrial oxygen consumption and oxidative phosphorylation efficiency. Oxidative phosphorylation efficiency of liver mitochondria from sham-operated control (open symbols) and CLP rats (close symbols). Succinate plus rotenone were used as substrate at different concentrations of exogenous ADP (100 μM, 20 μM, 5 μM, 1 μM) as well as oligomycin plus 100 μM ADP. Data are means ± SEM *p < 0.05 compared with matching sham-operated control group. Details of value and statistic significances are show in result section

The activities of the oxidative phosphorylation of liver mitochondria respiring on succinate (FADH-linked respiratory substrate) or pyruvate/malate (NADH-linked respiratory substrate) are shown in Fig. 2 and in supplementary data 1, respectively. For the sake of clarity, we do not put any significance symbols on our results of oxygen consumption and ATP production (Fig. 2 and Additional file 2). The relationships between the rates of ATP synthesis and oxygen consumption were linear in liver mitochondria regardless of the respiratory substrate used. For various exogenous amounts of ADP (100 μM, 20 μM, 5 μM, 1 μM), oxygen consumption of liver mitochondria energized with FADH-linked substrate (Fig. 2) was significantly reduced 24 h after the onset of CLP (87.45 ± 17.89, *p* = 0.0065; 66.02 ± 19.01, *p* = 0.0255; 42.72 ± 12.67, *p* = 0.029; 27.34 ± 6.99, *p* = 0.0077, *n* = 6) compared to the sham-operated control groups (173.62 ± 10.20, 92.90 ± 9.01, 57.14 ± 13.46, 45.34 ± 12.00, respectively, *n* = 6). Similar results were observed on mitochondria respiring on an NADH-linked substrate (online supplement data 2). However, differences were not significant in the early (6 h) or late (36 h) sepsis compared to mitochondria isolated from matching sham-operated control rats. Interestingly, the corresponding rates of ATP synthesis for the same amount of exogenous ADP added were lower at 24 h (144.51 ± 4.57, *p* = 0.0034; 82.02 ± 10.09, *p* = 0.0492; 47.47 ± 4.95 *p* = 0.0471; 12.84 ± 1.74, *p* = 0.0040) and 36 h (62.83 ± 12.71, *p* = 0.0446; 20.80 ± 3.43, *p* = 0.0020; 27.37 ± 3.32, *p* = 0.0274; 12.80 ± 7.50, *p* = 0.0080, *n* = 6) compared to matching sham-operated control rats at 24 h (265.31 ± 7.00, 121.06 ± 11.32, 93.33 ± 3.10, 38.83 ± 5.05, *n* = 6) and 36 h (144.16 ± 8.82, 117.83 ± 10.37, 81.16 ± 6.82, 32.16 ± 8.5, *n* = 6) but not at 6 h after the onset of CLP. These results indicate that oxidative phosphorylation efficiency, i.e., the amount of ATP synthesized per amount of oxygen consumed (ATP/O ratios), was not altered early after the onset of CLP (at least up to 24 h) but decreased after 36 h of sepsis induction. This is further illustrated by the relationships between the rates of ATP synthesis and oxygen consumption of mitochondria isolated from CLP and corresponding control rats, which were superimposed at 6 and 24 h after the onset of surgery. By contrast, the linear relationship shifted to the right at 36 h (Fig. 2c), indicating that for a given amount of oxygen consumed, less ATP was produced by liver mitochondria.

### Effect of sepsis on mitochondrial cytochrome c oxidase and ATP synthase proteins

**Fig. 3:**
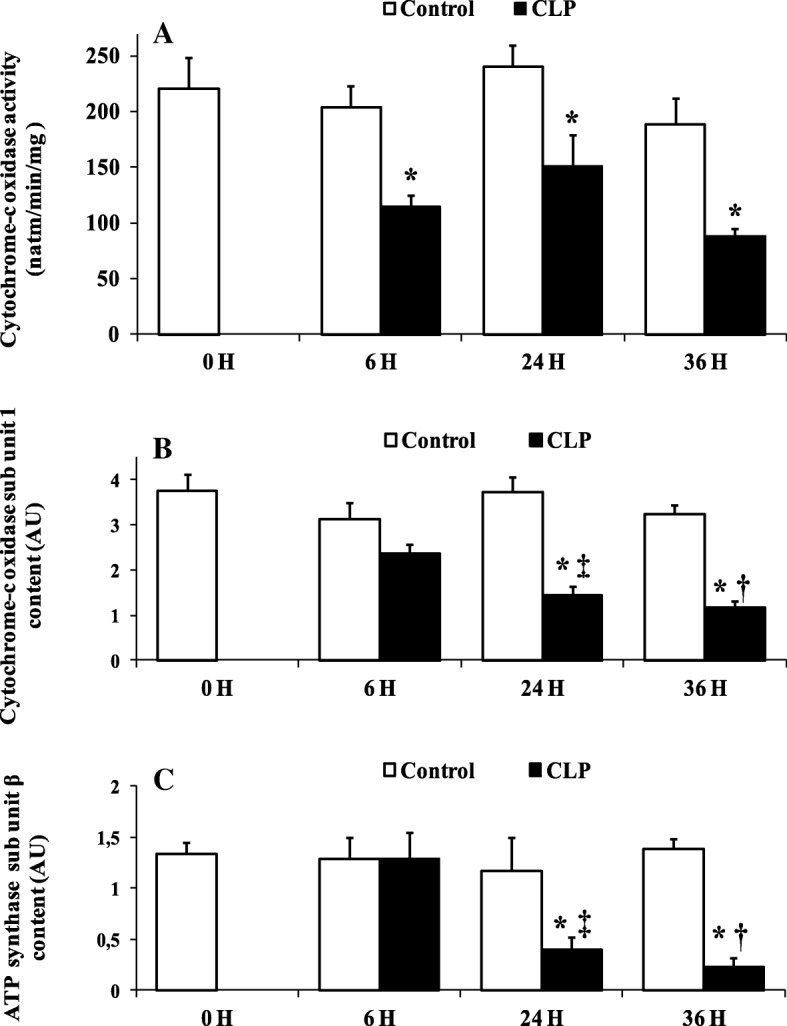
Cytochrome-c oxidase activity and sub unit 1 content, and ATP synthase sub unit β content. **a** Liver mitochondrial cytochrome c oxidase maximal activity of controls groups (naïve and sham-operated control) (white bars) and CLP groups (black bars). Data are means ± SEM. *p < 0.05 versus sham-operated and naïve rats from five animals per group determined by surgical procedure and following period (0, 6, 24, 36 h). Western blot analysis of liver mitochondrial cytochrome-c oxidase sub unit 1 (**b**), and ATP synthase sub unit β (**c**) in controls groups (white bars) and CLP groups (black bars). Data are means ± SEM. *p < 0.05 versus matching sham-operated control rats; ‡*p* < 0.05 compared 6 h CLP and 24 h CLP; †*p* < 0.05 compared 6 h CLP and 36 h CLP, from five animals per group determined by surgical procedure and following period (6, 24, 36 h)

The lower rate of oxidative phosphorylation observed above was associated with a decrease in cytochrome c oxidase activity and in cytochrome c oxidase and ATP synthase contents (Fig. 3). The activity of cytochrome c oxidase was negatively affected early by the septic shock (Fig. 3a), being 43% lower 6 h post-CLP (115.80 ± 9.40, *p* = 0.0383, *n* = 6) compared to sham-operated control rats (204.40 ± 19.97, *n* = 6) (Fig. 3a). The reduction of activity of cytochrome c oxidase was found in all CLP groups and was associated with a concomitant decrease in the amount of mitochondrial cytochrome c oxidase subunit 1 (CcO1) (Fig. 3b). The maximal reductions of cytochrome c oxidase activity (− 53%) (88.00 ± 7.01, *p* = 0.033, *n* = 6) (Fig. 3a) and content (− 35%) (1.18 ± 0.28, *p* = 0.028, *n* = 5) (Fig. 3b) were observed at the same time, 36 h after CLP, compared to matching sham-operated (188.60 ± 24.7 *n* = 6, 3.34 ± 1.34 *n* = 5). The amount of ATP synthase subunit β was also significantly decreased at 24 h (− 55%) (0.33 ± 0.11, *p* = 0.047, *n* = 5) and 36 h (− 63%) (0.17 ± 0.05, *p* = 0.047, *n* = 5) after CLP compared to matching sham-operated rats at 24 h (1.17 ± 0.43, *n* = 5) and 36 h (1.38 ± 1.10, *n* = 5) (Fig. 3c).

### Effect of sepsis on inner membrane permeability to protons and mitochondrial oligomycin-insensitive respiration

**Fig. 4:**
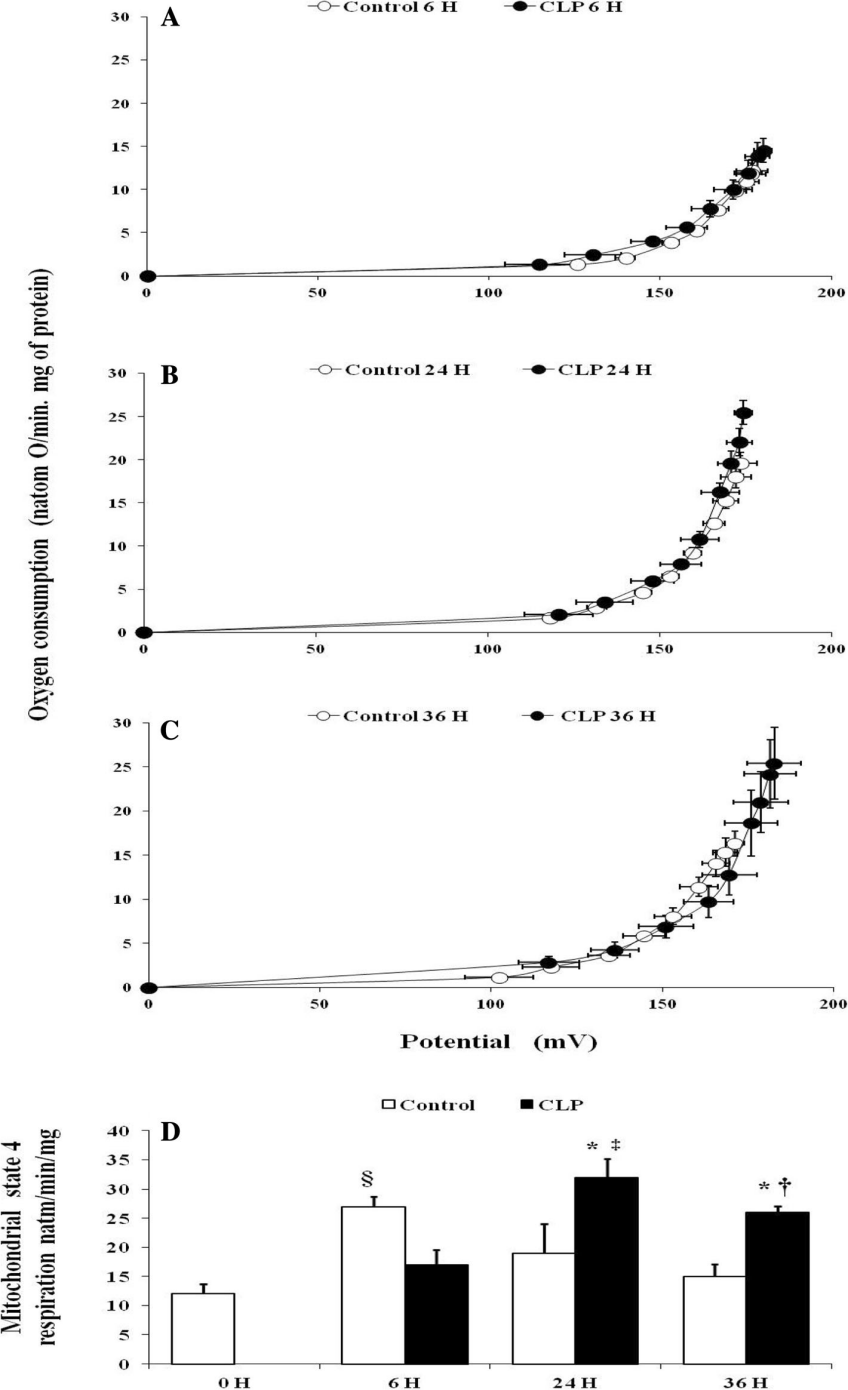
Mitochondrial proton permeability and basal oxygen consumption. Basal proton leak in liver mitochondria from matching sham-operated control (open symbols *n* = 6) and CLP rats (closed symbols *n* = 6), at 6, 24, 36 h after surgery (**a**–**c**). Respiration and membrane potential were measured in the presence of oligomycin, nigericin, rotenone, and succinate as described in “Materials and methods” section. Sequential addition of malonate up to 3 mM was performed to induce variations in membrane potential. Values are means ± SEM from six different rats per group. **d** Liver mitochondria oligomycin-insensitive respiration of control (naïve and matching sham-operated) (white bars), and CLP rats (black bars). Data are means ± SEM. *p < 0.05 versus sham-operated and naïve rats; ‡*p* < 0.05 compared 6 h CLP and 24 h CLP; †*p* < 0.05 compared 6 h CLP and 36 h CLP, §*p* < 0.05 compared matching sham- operated control and naïve (control 0 h) rats; from six animals per group determined by surgical procedure and following period (0, 6, 24, 36 h)

The mitochondrial membrane permeability to proton as reveled by the relationship between membrane potential and mitochondrial oligomycin-insensitive respiration was unaffected by septic shock after 6, 24, or 36 h of CLP (Fig. 4a–c). However, the maximal rate of oligomycin-insensitive respiration, the highest point to the right in the relationship curve, was significantly higher in the CLP group at 24 h (+ 48%) (32.66 ± 5.72, *p* = 0.017, *n* = 6) (Fig. 4b, d) and 36 h (+ 37%) (25.80 ± 1.09, *p* = 0.029, *n* = 6) (Fig. 4c, d) after the onset of CLP than matching sham-operated rats at 24 h (19.50 ± 1.58, *n* = 6) and 36 h (15.60 ± 1.02, *n* = 6).

### Effect of sepsis on mitochondrial biogenesis factors

**Fig. 5:**
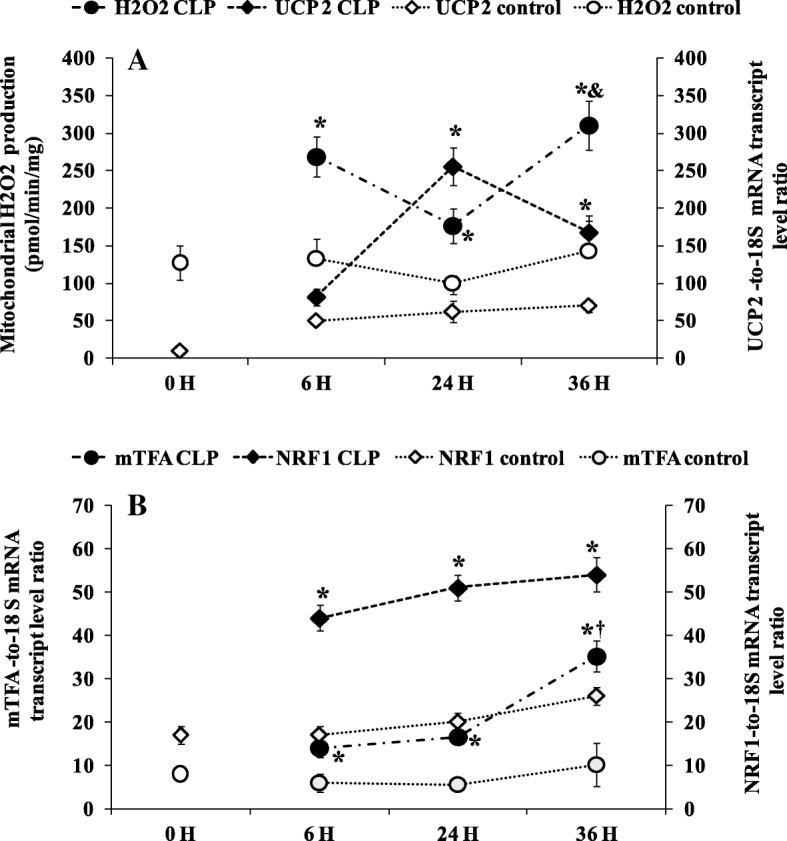
**a** Relation between liver uncoupling protein 2 (UCP 2) mRNA gene expression and liver mitochondrial H_2_O_2_ production. UCP 2 mRNA was determined by real-time quantitative PCR in the liver of control groups (naïve and sham-operated) (white symbols) white symbols) and CLP groups (black symbols). Data are means ± SEM. *p < 0.05 versus sham-operated and naïve rats; &*p* < 0.05 compared 24 h CLP and 36 h CLP, from five animals per group determined by surgical procedure and following period (0, 6, 24, 36 h). Mitochondrial H_2_O_2_ production was measured in the presence of succinate without rotenone. *p < 0.05 versus sham-operated and naïve rats; &*p* < 0.05 compared 24 h CLP and 36 h CLP, from six animals per group determined by surgical procedure and following period (0, 6, 24, 36 h). **b** Mitochondrial transcription factor A (mTFA) and nuclear respiratory factor 1 (NRF 1) mRNA level were determined by real-time quantitative PCR in the liver of control groups (naïve and sham-operated) (white symbols) and CLP groups (black symbols). Data are means ± SEM. *p < 0.05 versus sham-operated and naïve rats and †*p* < 0.05 compared 6 h CLP and 36 h CLP, from five animals per group determined by surgical procedure and following period (0, 6, 24, 36 h)

Mitochondrial biogenesis factor mRNA levels (mTFA and NRF1) were higher during the time course of septic shock compared to sham-operated control rats and naïve (0-h control) rats. Among CLP groups, we found significantly higher mitochondrial transcription factor A (mTFA) gene expression at 24 h (16.56 ± 4.83, *p* = 0.014, *n* = 5) and 36 h (35.17 ± 3.54, *p* = 0.019, *n* = 5) compared to matching sham-operated rats at 24 h (5.51 ± 1.50 *n* = 5) and 36 h (10.10 ± 5.00, *n* = 5) (Fig. 5b).

### Effect of sepsis on mitochondrial ROS generation and UCP2 gene expression

Figure 5a shows the effect of sepsis on mitochondrial ROS (H_2_O_2_) generation and UCP2 gene expression. On the whole, mitochondrial H_2_O_2_ generation was higher in CLP groups than sham-operated and naïve rats. Further inside, the amount of H_2_O_2_ was higher at 6 h (269 ± 23, *p* = 0.0398, *n* = 6) and 36 h (310.60 ± 33.58, *p* = 0.0087, *n* = 6) than matching sham-operated rats at 6 h (162.60 ± 22.69, *n* = 6) and 36 h (163.80 ± 40.36, *n* = 6). Among the CLP groups, mitochondria of 36-h rats produced higher H_2_O_2_ (+ 43%, *p* = 0.0158, *n* = 6) than 24-h rats. The pattern of ROS generation was the opposite of that of UCP2 gene expression, as shown by Fig. 5a. UCP2 mRNA level was higher at 24 h (255.16 ± 23.85, *p* = 0.0106, *n* = 5) and 36 h (166.50 ± 22.71, *p* = 0.0176, *n* = 5) compared to matching sham-operated control rats at 24 h (62.40 ± 14.62 *n* = 5) and 36 h (69.60 ± 8.85 *n* = 5). However, the difference between the 24- and 36-h CLP groups did not reach significance.

## Discussion

Our results provide evidence of impairment of liver mitochondrial oxidative phosphorylation. In CLP rats, we found a decrease in ATP synthesis and oxygen consumption at 24 h following CLP. Uncoupling of oxidative phosphorylation occurring at 36 h was associated with a decrease in ATP synthase subunit β content and cytochrome c oxidase activity and content (slip mechanism). The increase in mitochondrial oligomycin-insensitive respiration was not associated with changes in mitochondrial inner membrane permeability (indicating no change in mitochondrial proton leak). This increase in mitochondrial respiration non-coupled with phosphorylation was associated with increased UCP2 mRNA at 24 h and might be a mechanism to limit ROS generation but does not help at increase mitochondrial efficiency.

Increasing evidence suggests a relevant role for impaired mitochondrial oxidative phosphorylation and consequent excessive ROS generation in sepsis-induced organ injury [9, 10, 21, 22, 31]. This alteration of mitochondrial oxidative phosphorylation could be the consequence of at least a twofold mechanism. Dissipation of the mitochondrial electrochemical gradient through membrane proton leakage and/or the inability of mitochondria to bring into play a compensatory mechanism of impaired mitochondria respiratory chain and oxidative phosphorylation. Some of these compensatory mechanisms were investigated in this study. Consistent with previous studies [11, 32, 33], we observed a decrease in oxygen and ATP fluxes in septic groups. The decrease in oxygen consumption was associated with a proportional decrease in ATP generation 24 h after the onset of CLP, whereas at 36 h the depletion in ATP synthesis was not associated with a proportional drop in oxygen consumption, indicating a reduction in the mitochondrial oxidative phosphorylation efficiency (ATP/O) (i.e., decreased ATP generation while mitochondrial oxygen and substrates are available in steady-state conditions). Mechanistically, the decrease in mitochondrial efficiency could be the consequence of an alteration of the energy transduction system (respiratory chain at the level of complex 1, electrochemical gradient, ADP phosphorylation) [9, 22] or regulatory mechanisms of oxidative phosphorylation (i.e., redox slip (intrinsic uncoupling of mitochondrial respiratory chain) and/or leak (increased in inner membrane permeability to protons)). In particular, cytochrome c oxidase activity and/or stoichiometry are believed to control and adjust oxidative phosphorylation in stress conditions [20, 21, 34]. Indeed, several studies provide evidence that when the activity and content of cytochrome c oxidase decrease (redox slip) [34] or increase [35, 36], the mitochondrial efficiency (ATP/O) moves in the opposite way. The present study shows that sepsis decreased the content and activity of cytochrome c oxidase, in line with previous studies [33, 37–39]. However, contrary to what could be expected, this was not associated with an increase in oxidative phosphorylation efficiency ATP/O, which could have compensated for energy production (ATP generation) from mitochondrial respiratory chain oxidative loss. This result reinforces and extends previous data [25] on the failure of the mitochondrial oxidative phosphorylation system to adjust its intrinsic coupling efficiency in early septic shock. Furthermore, in line with previous findings that ROS [40], sepsis [41], or endotoxemia [42] negatively affects ATP synthase activity, the late inhibition and depletion of ATP synthase subunit β observed in the present study 36 h after the onset of CLP may also explain mitochondrial uncoupling of ATP production from oxygen consumption. Consequently, mitochondria would not meet cellular energy requirements in the early septic shock, a situation that worsens and leads to organ failure once mitochondrial efficiency starts to decrease while mitochondrial activity remains low. Mitochondria matches respiration to ATP synthesis by building up an electrochemical gradient of protons [43]. A dissipation of membrane potential (e.g., proton leak activity) can cause uncoupling of mitochondrial ATP synthesis from respiration [19, 20, 44]. Changes in the electrochemical gradient through the increase in the inner mitochondrial membrane permeability to protons are a well-known regulatory mechanism of oxidative phosphorylation efficiency [19, 20, 44]. This could be driven by a modification of an intrinsic property of the inner membrane (e.g., fatty acyl membrane composition) or activation of uncoupling proteins (UCPs) following an increase in ROS generation [19, 20, 44]. Some reports have suggested that a UCP-mediated uncoupling by an increasing inner membrane permeability to protons can decrease ATP synthesis [45] and impair cardiac efficiency under septic conditions [45, 46]. The present study showed no alteration of mitochondrial conductance to protons in spite of an increase in non-coupled (with phosphorylation) respiration (Fig. 4d) and increasing level of UCP2 mRNA in the time course sepsis. This result suggests that dissipation of membrane potential is not a cause of the mitochondrial oxidative phosphorylation uncoupling observed at 36 h following CLP. Together, the increases in non-coupled respiration and UCP2 mRNA observed at 24 h after the onset of CLP were effective to limit ROS generation. Indeed, mitochondrial ROS generation decreased 24 h after the onset of CLP, when UCP2 mRNA content was at the highest. Then, 12 h later, at the 36th hour from CLP, mitochondrial ROS generation increased again, inversely to the decrease in UCP2 mRNA concentration. Published evidence shows that UCP2 mRNA peaks from 6 to 24 h after CLP in heart and kidney [45–47] or endotoxic shock in liver cells [48] through proinflammatory cytokine activation. Even though we did not assess the uncoupling activity of UCP2 or do a protein assay, the variation of ROS generation observed was somehow inversely related to the expression of the UCP2 gene (Fig. 5a). Our study also shows that the decrease at the same time in cytochrome c oxidase activity and content and in UCP mRNA at 36 h after the onset of septic shock resulted in increased ROS generation (Figs. 2 and 5a). These results suggest that cytochrome c oxidase might be responsible for the higher mitochondrial ROS generation observed in the septic shock groups. Indeed, partial suppression of cytochrome c oxidase activity induces an increase in ROS generation [18], whereas an increased activity of this mitochondrial enzyme results in decreased ROS production at the level of mitochondria [36]. As a rational consequence, the artificial increase in cytochrome c oxidase activity by cytochrome c infusion has been reported to restore mitochondrial oxygen consumption [49, 50] and decrease ROS generation.

A limit of our study is the ex vivo measurement of oxygen consumption at non physiological level of oxygen. However, Clark-type electrode has been used over the 50 years and has led to an intimate understanding of mitochondrial respiratory function.

## Conclusion

During the septic shock induced by CLP, despite a compensatory increase in mitochondrial biogenesis factors [51], liver mitochondrial metabolism remained profoundly altered. This suggests that all of the functional compensation mechanisms reported in the present study (upregulation of mitochondrial biogenesis factors, slipping reactions at cytochrome c oxidase) were not strong enough to reverse the mitochondrial alterations and thus failed to protect the liver against excessive mitochondrial ROS generation.

## Additional files

Additional file 1: Primers used for the determination by real-time PCR of mRNA concentrations. (DOC 40 kb)

Additional file 2: Mitochondrial oxygen consumption and oxidative phosphorylation efficiency. Oxidative phosphorylation efficiency of liver mitochondria from sham-operated control (open symbols) and CLP rats (close symbols). Pyruvate (5 mM) plus malate (2.5 mM) were used as substrate at different concentrations of exogenous ADP (100 μM, 20 μM, 5 μM, 1 μM) as well as oligomycin plus 100 μM ADP. Data are means ± SEM * *p* < 0.05 compared with matching sham-operated control group. Details of value and statistic significances are show in result section. (DOC 75 kb)

## Abbreviations

ALT: Alanine amino transferase
ATP: Adenosine triphosphate
CcO1: Cytochrome c oxidase subunit 1
CLP: Cecal ligation and puncture
FCCP: Carbonyl cyanide-p-tri-fluoro-methoxy-phenyl-hydrazone
mTFA: Mitochondrial transcription factor A
NRF 1: Nuclear respiratory factor 1
ROS: Reactive oxygen species
TBS: Tris-buffered saline
TMPD: N,N,N′,N′-tetramethyl-1,4-benzenediamine dichloride
TPMP: Methyl-tri-phenyl-phosphonium
UCP: Uncoupling protein
