# Supplementary material for: Time course of liver mitochondrial function and intrinsic changes in oxidative phosphorylation in a rat model of sepsis
Source: Intensive Care Med Exp. 2018 Sep 5;6:31. doi: 10.1186/s40635-018-0197-y (PMC6125261; doi:10.1186/s40635-018-0197-y)
Supplement: Supplementary file 1 — Primers used for the determination by real-time PCR of mRNA concentrations. (DOC 40 kb) [file 40635_2018_197_MOESM1_ESM.doc]

**Additional file 1**

Primers used for the determination by real-time PCR of mRNA concentrations:

| Gene name  (gene bank reference) | Forward primer | Reverse primer | Size (bp) |
| --- | --- | --- | --- |
| Nuclear Respiratory Factor 1  (NRF 1)  (NM_031326.1) | GCGGGAGGACCTTCTGTATG | CAGGGTTGCTGAAGGTCTGT | 126 |
| Mitochondrial transcription factor A  (mTFA)  (NM_031326.1) | TCATGACGAGTTCTGCCGTT | CTTCACAAACCCGCACGAAA | 145 |
| IL 1 β  (NM_031512.2) | GGCTGACAGACCCCAAAAGA | TTGTCGAGATGCTGCTGTGA | 92 |
| TNF-α  (NM_012675) | CGAGATGTGGAACTGGCAGA | CGATCACCCCGAAGTTCAGT | 147 |
| Uncoupling Protein 2  (UCP 2)  (NM_019354.2) | CCTCCCTTGCCACTTCACTT | AGGAAGGCATGAACCCCTTG | 190 |
| 18S  (NR_046237.1) | TGAGGCCATGATTAAGAGGG | AGTCGGCATCGTTTATGGTC | 190 |
