# Supplementary material for: Time course of liver mitochondrial function and intrinsic changes in oxidative phosphorylation in a rat model of sepsis
Source: Intensive Care Med Exp. 2018 Sep 5;6:31. doi: 10.1186/s40635-018-0197-y (PMC6125261; doi:10.1186/s40635-018-0197-y)
Supplement: Supplementary file 2 [file 40635_2018_197_MOESM2_ESM.doc]

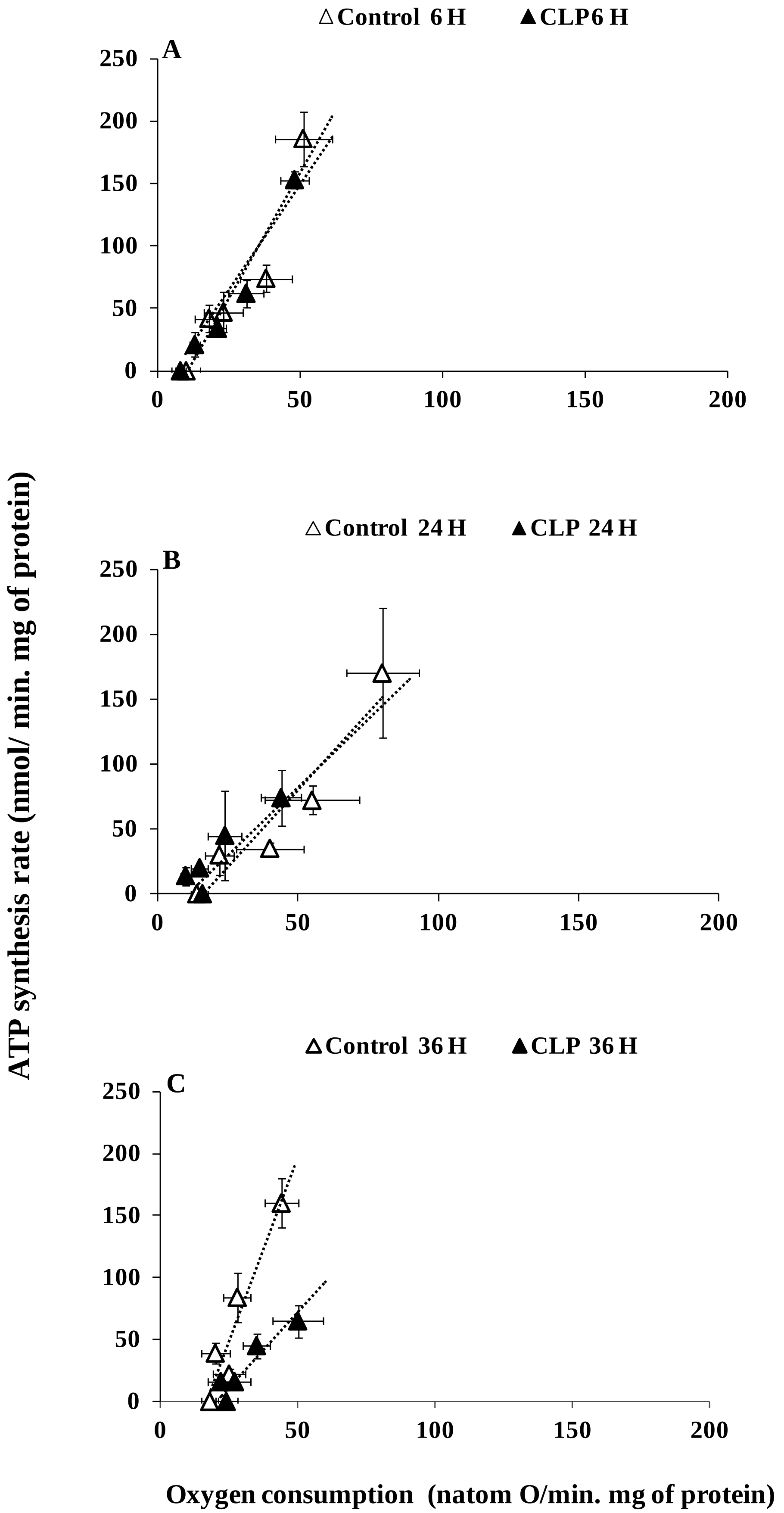

△Control 6 H ▲CLP6 H
A
△Control 24 H ▲CLP 24 H
B
△Control 36 H ▲CLP 36 H
C
ATP synthesis rate (nmol/ min. mg of protein)
Oxygen consumption (natom O/min. mg of protein)
